# Expressions of ABCG2, CD133, and Podoplanin in Salivary Adenoid Cystic Carcinoma

**DOI:** 10.1155/2014/132349

**Published:** 2014-04-06

**Authors:** Wuwei Li, Ryo Tamamura, Bo Wang, Qigui Liu, Han Liu, Tingjiao Liu, Naoki Katase, Jing Xiao, Hitoshi Nagatsuka

**Affiliations:** ^1^Medical College of Stomatology, Dalian Medical University, 9 Western Section, Lvshun South Street, Dalian 116044, China; ^2^Department of Histology, Nihon University School of Dentistry at Matsudo, 2-870-1 Sakaemachi-nishi, Matsudo 271-8587, Japan; ^3^Comprehensive Transplant Center, Department of Surgery, Feinberg School of Medicine, Northwestern University, 300 E. Superior Street, Chicago, IL 60611, USA; ^4^Department of Health Statistics, Dalian Medical University, 9 Western Section, Lvshun South Street, Dalian 116044, China; ^5^Department of Oral Pathology and Medicine, Graduate School of Medicine, Dentistry and Pharmaceutical Sciences, Okayama University, 2-5-1 Shikata-cho, Okayama 700-8525, Japan

## Abstract

Adenoid cystic carcinoma (ACC) is one of the most common salivary gland malignant tumors with a high risk of recurrence and metastasis. Current studies on cancer stem cells (CSCs) have verified that CSCs are the driving force behind tumor initiation and progression, suggesting that new cancer therapies may be established by effectively targeting and killing the CSCs. The primary goal of this study is to investigate the expression patterns of ABCG2, CD133, and podoplanin in ACC of minor salivary glands by immunohistochemistry analysis. We found that ABCG2 was weakly expressed in normal looking salivary gland tissues. A significant upregulation of ABCG2 expression in ACC was observed with a similar expression pattern of Ki-67. CD133 was detected in apical membrane of epithelial cells and podoplanin was expressed positively in myoepithelial cells of both normal looking tissue and ACC. However, no significant difference was found of the expression pattern of CD133 and podoplanin between normal looking tissues and ACC. Our observations suggest that CSCs may exist in quiescent cells with ABCG2 positive staining, which are surrounded by cells with positive expression of ABCG2 and Ki-67 in ACC, and costaining with ABCG2 and Ki-67 may help predict the location of CSCs.

## 1. Introduction

Adenoid cystic carcinoma (ACC) is one of the most common malignant neoplasms that affects either the major or minor salivary glands of the oral cavity with high risk of recurrence and metastasis [1]. About 50% of ACCs occur in the hard palate and rarely take place in other intraoral sites including the lower lip, retromolar/tonsillar pillar region, sublingual gland, and buccal mucosa [2]. Currently, surgery and radiotherapy are the most effective treatments, but the outcomes remain unsatisfying [3]. In many cases, ACC shows an indolent clinical course with a considerable risk of local relapse and late distant metastases to lung or bone, which occasionally causes fatal outcome after the first episode [3].

The hypothesis of cancer stem cells states that the tumor formation and growth likely depend on a small subset of tumor cells, known as cancer stem cells (CSCs) or tumor-initiating cells (TICs), which might be the origin of neoplasia [4]. CSCs have been isolated from some types of solid tumors, such as breast, brain, prostate, melanoma, colon, pancreas, head/neck, liver, and gastric cancers [5–8], and they are recognized to be more resistant to radiotherapy and chemotherapy and additionally invasive than normal tumor cells. If CSCs can be targeted by specific CSC markers and killed efficiently, the outcomes of current clinical cancer treatments may be improved [9]. Several putative stem cell markers, such as CD44, CD24, CD166, EpCAM, ABCG2, CD133, and podoplanin, are commonly studied in identifying and isolating the CSCs from the solid tumors; however, subsequent studies demonstrated that the choice of CSC markers differs among cancer originations [10–15].

ATP-binding cassette, subfamily G, member 2 (ABCG2) is a member of the superfamily of ATP-binding cassette transporter proteins. Immunohistochemical analysis showed that ABCG2 was expressed ubiquitously in normal and tumor tissues [16]. Zhou et al. [17] found that the expression of* ABCG2* gene is an important determinant and a molecular marker for side population-enriched stem cells (SP cells), and this feature was considered to be related to CSCs [18]. Kim et al. also reported that ABCG2 is highly expressed in SP cells and its expression is strongly correlated with SP phenotype [10]. ABCG2 can be expressed in stem cells isolated from both normal and tumor tissues, which further indicates its essential role in stem cell biology [19].

CD133 (prominin-1), a cell-surface glycoprotein carrying five transmembrane domains, was firstly identified as a marker for a subpopulation of CD34-positive haematopoietic stem cells which are derived from human fetal liver, fetal bone marrow, and peripheral blood [20]. CD133 was initially regarded as a specific marker of hematopoietic stem cell and later found in neural stem cells, epidermal stem cells, and endothelial progenitor cells (EPCs) [17, 21, 22]. Previous studies have identified and isolated a putative CSC population from the brain [23], prostate [24], liver [25], lung [26], and colon [27] cancers using CD133 antibodies. In oral CSC-like cells isolated from primary tumor cells and cell lines, scientists found an improved migration and tumorigenicity behavior of these cells with a higher CD133 expression level [28]. CD133-positive cells also showed an enhanced clonogenicity, invasiveness, and tumorigenicity compared to those CD133-negative cells [29].

Podoplanin is a mucin-like transmembrane glycoprotein that is highly and specifically expressed in lymphatic endothelial cells of normal tissues and tumors [30]. Recent studies have reported that podoplanin-positive cells in tumors act as mediators of metastasis and invasion [31–33]. Podoplanin is also reported as a potential CSC marker for squamous-cell carcinoma (SCC). Atsumi et al. found that A431 cells (a SCC cell line) consisted of both podoplanin-positive and podoplanin-negative cells, in which podoplanin-negative cells only produced podoplanin-negative cells but podoplanin-positive cells generated both podoplanin-positive and -negative cells, and podoplanin-positive cells showed higher tumorigenicity [34]. Rahadiani et al. reported that the esophageal squamous-cell carcinoma (ESCC) showed defective invasion and tumorigenic activities after the podoplanin expression were knocked down in esophageal [35].

Relative studies in identifying CSCs markers in ACC are still in the early stage. In this study, we examined the expression patterns of ABCG2, CD133, and podoplanin in ACC of minor salivary glands by immunohistochemical analysis. Meanwhile, Ki-67, a nuclear protein associated with cellular proliferation, was used to evaluate the proliferation of the tumor cells.

## 2. Materials and Methods

### 2.1. Tissue Samples

Archival paraffin-embedded tissue blocks of 25 ACC cases (9 men, 16 women; mean age: 60.72 years; range: 32–78 years) were randomly selected. Ten of these samples included normal looking tissues. These samples were diagnosed at Okayama University Hospital Surgical Pathology Unit (Okayama, Japan) from 1980 to 2005. All specimens were fixed with 10% neutral buffered formalin and embedded in paraffin. Histological diagnosis was carried out by routine hematoxylin and eosin (H and E) staining, according to World Health Organization (WHO) histological typing of ACC. Clinical and histopathological characteristics of the patients are shown in Table 1. Ten normal looking salivary gland tissues were used as the control group in this study. Informed consents were obtained from the patients and the experiment was approved by the Research Ethics Board of Okayama University.

### 2.2. Immunohistochemistry Staining

Antibodies against ABCG2 (BXP-21) (1 : 50; mouse monoclonal; Abcam, Cambridge, UK), CD133 (1 : 300; rabbit polyclonal; Abcam), podoplanin (1 : 50; mouse monoclonal; DAKO, Carpinteria, CA) and Ki-67 (1 : 50; mouse monoclonal; DAKO) were used in this study.

For immunohistochemical staining, serial sections of 4 *μ*m thickness were deparaffinized in xylene and rehydrated in decreasing concentrations of ethanol. Endogenous peroxidase activity was blocked by immersing the sections in 3% H_2_O_2_ with methanol for 30 min. For antigen retrieval, sections were boiled in 10 mmol/L citrate buffer (pH 6.0) for 15 min in a pressure cooker. After treatment with protein block serum at room temperature, sections were covered with primary antibodies and incubated at 4°C overnight. For secondary antibody, DAKO Cytomation Envision+System-HRP kit (AEC) was used according to the manufacturer's instructions. Antibody reactions were stained with 3,3′-diaminobenzidine and counterstained with hematoxylin. For the negative control, sections were incubated with PBS instead of the primary antibodies.

### 2.3. Assessment Standard

ABCG2, CD133, and podoplanin were detected in the cytoplasm and/or cell membrane. Ten areas were selected randomly in each section under high power magnification (×400). Also, 100 cells were selected randomly in each area so that a total of 1000 cells were analyzed for counting the positive cells. Positive staining intensity for cancer stem cell markers was classified as negative: − (<30%), weak: + (30–49%), moderate: ++ (50–74%), and strong staining: +++ (≥75%). For Ki-67 staining, the nucleus was stained with brown color and positive staining intensity was classified as negative: − (<5%), weak: + (5–9%), moderate: ++ (10–14%), and strong staining: +++ (≥15%). No false positive or negative staining was encountered in the positive or negative controls, respectively.

### 2.4. Statistical Analysis

Statistical analyses were performed with* SPSS* version 18.0 for Windows. Wilcoxon test was used to determine the intensity of marker expression and* Fisher's* exact test was used to determine the positive percentage of tumor cells and clinicopathological characteristics. Correlations between different markers and Ki-67 expression intensities in different locations of ACC and normal looking tissues were assessed by Spearman's rank correlation analysis. All tests were two-tailed and the differences were considered statistically significant when *P* < 0.05.

## 3. Results

### 3.1. Expression of ABCG2, CD133, Podoplanin, and Ki-67 in Normal Looking Tissues

**Figure 1:**
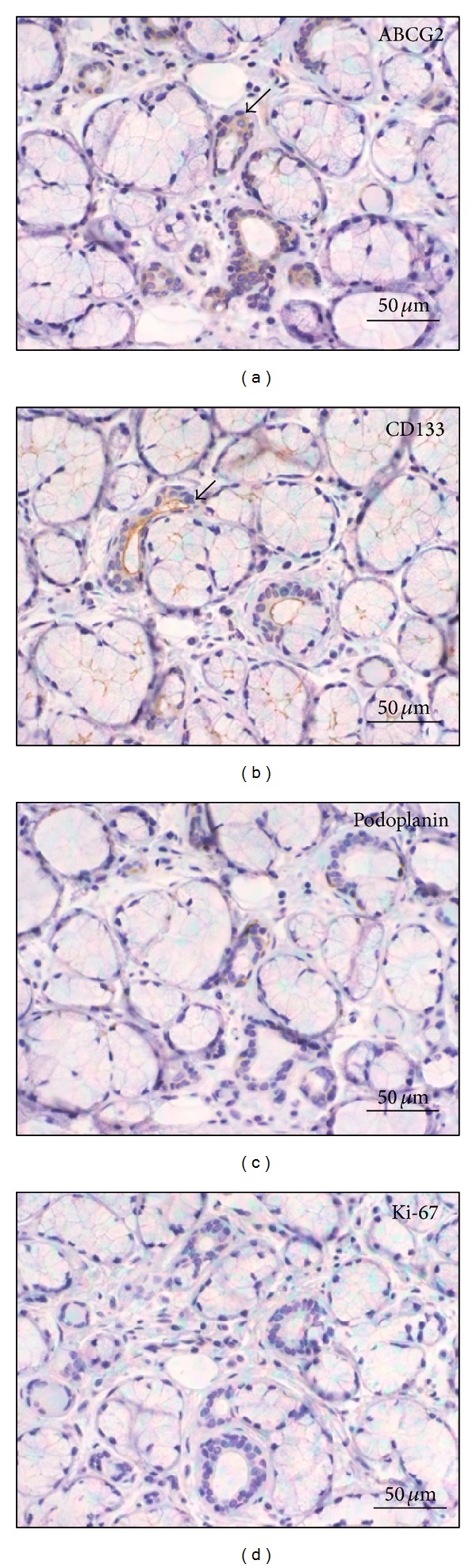
Expression of (a) ABCG2, (b) CD133, (c) podoplanin, and (d) Ki-67 in normal looking tissues.

Immunopositivity for ABCG2 was observed in the cytoplasm and cell membrane of acinar epithelium cells, ductal cells, and myoepithelial cells in 6 cases and the staining intensities were weak in all 6 samples (Figure 1(a); Table 2). A small number of ABCG2-positive cells were present in the capillary endothelium. CD133-positive expressions were localized only in apical membrane of acinar epithelial cells and ductal cells in all 10 samples (Figure 1(b)) in which 5 cases showed moderate staining intensity and 5 cases showed strong intensity. Podoplanin-positive expressions were found in the cytoplasm and cell membrane of myoepithelial cells in just 1 case with weak staining intensity (Figure 1(c)). Expression of Ki-67 was negative in all 10 cases (Figure 1(d)).

### 3.2. Expression of ABCG2, CD133, Podoplanin, and Ki-67 in ACC

*ABCG2.* ABCG2-positive stainings could be detected in 23 cases including 1 case of weak staining intensity, 7 cases of moderate intensity, and 15 cases of strong* intensity*, and the positive expression percentage was 92% (*P* = 0.043) (Table 2). The tumor cells showed strong ABCG2 staining in the invasive front of cancer nests and the intensity in peripheral zone was stronger than the center zone. The intensity of ABCG2 staining was significantly stronger in ACC than in the normal looking tissues (*P* = 0.000).

**Figure 2:**
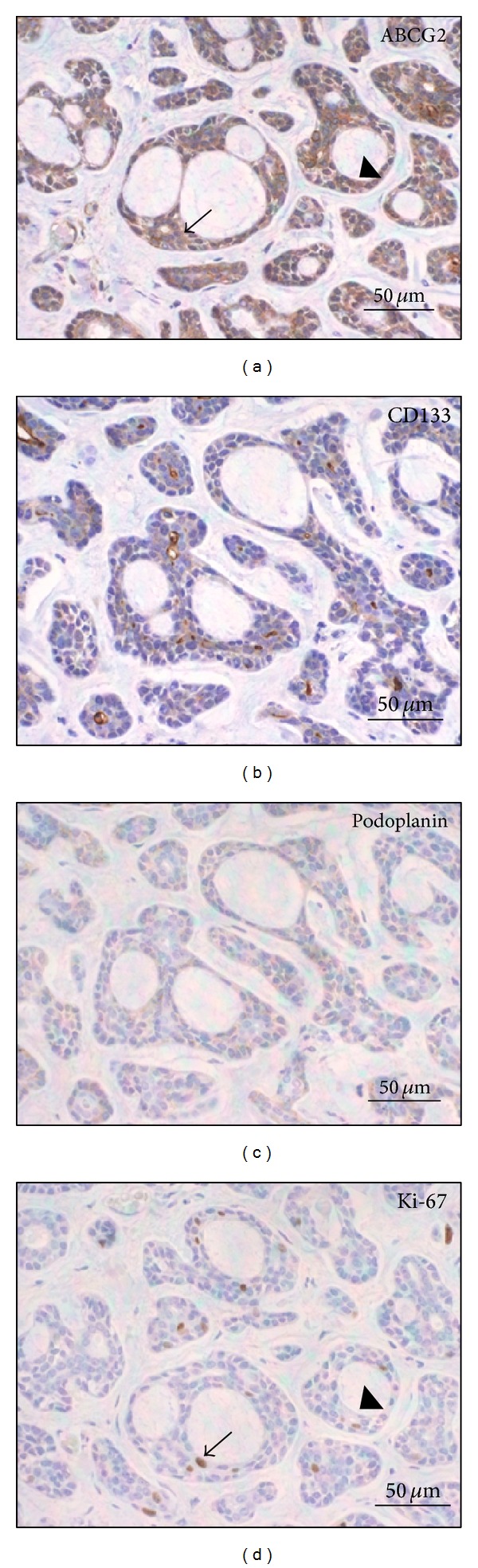
Expression of (a) ABCG2, (b) CD133, (c) podoplanin, and (d) Ki-67 in ACC of cribriform pattern.

**Figure 3:**
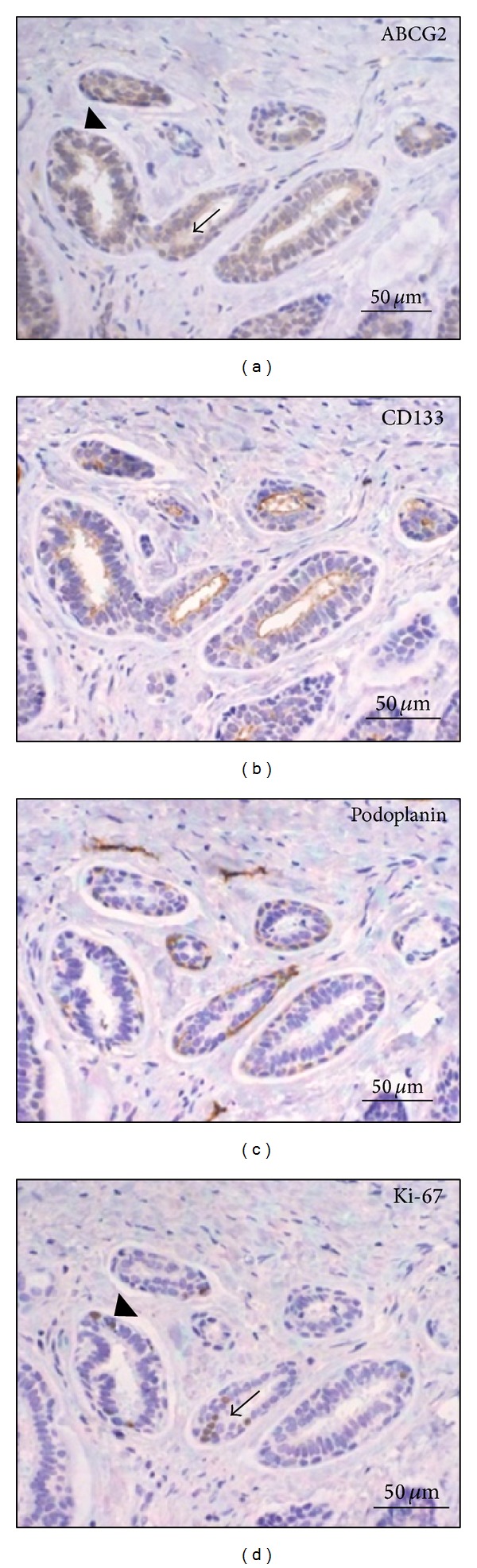
Expression of (a) ABCG2, (b) CD133, (c) podoplanin, and (d) Ki-67 in ACC of tubular pattern.

**Figure 4:**
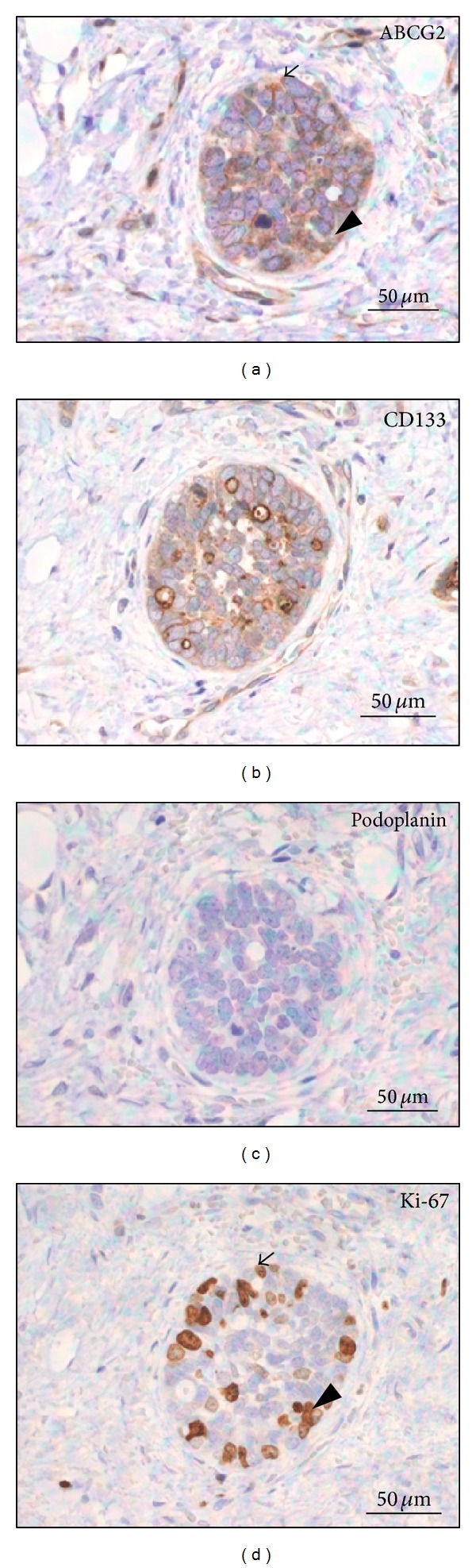
Expression of (a) ABCG2, (b) CD133, (c) podoplanin, and (d) Ki-67 in ACC of solid pattern.

In the cribriform pattern of ACC, ABCG2 staining was observed on both cytoplasm and membrane of the epithelial cells. The highest intensity of ABCG2 staining was mainly localized in the periphery of cancer nests (Figure 2(a)). A similar pattern of ABCG2-positive staining in stromal vascular endothelial cells was also observed in the normal looking salivary gland tissues. In tubular pattern, ABCG2 was expressed in glandular epithelial cells (Figure 3(a)). In solid pattern, the myoepithelial cells were diffusely positive for ABCG2, and the highest intensity of ABCG2 staining was found focally in both periphery and center of cancer nests (Figure 4(a)).**

*CD133.* CD133-positive cells were detected in 25 cases (1 case with weak intensity, 12 cases with moderate intensity, and 12 cases with strong intensity) but limited in the apical membrane of the luminal cells in cribriform (Figure 2(b)) and tubular (Figure 3(b)) patterns. The contents in the ductal structures and pseudocyst spaces were positive for CD133. In solid pattern, positive expression was mainly diffused among the dense myoepithelial cells (Figure 4(b)). There was no significant difference of CD133 intensity or positivity between ACC and normal looking tissues (*P* = 0.835  and  *P* = 1.000, resp.).**

*Podoplanin.* Podoplanin expressions were detected in 6 cases of ACC including 4 cases with weak staining intensity, 1 case with moderate intensity, and 1 case with strong* intensity*, and the average positive percentage was 24% (*P* = 0.644). The intensity in ACC showed no significant difference compared with the normal looking tissues (*P* = 0.332). Podoplanin can be only observed in myoepithelial cells and lymphatic endothelial cells. In most of the samples of cribriform pattern, podoplanin showed negative staining (Figure 2(c)). In tubular pattern of ACC, expressions of podoplanin were mainly detected in myoepithelial cells but not in glandular epithelial cells (Figure 3(c)). Negative podoplanin expressions were detected in all solid pattern samples (Figure 4(c)).

**Figure 5:**
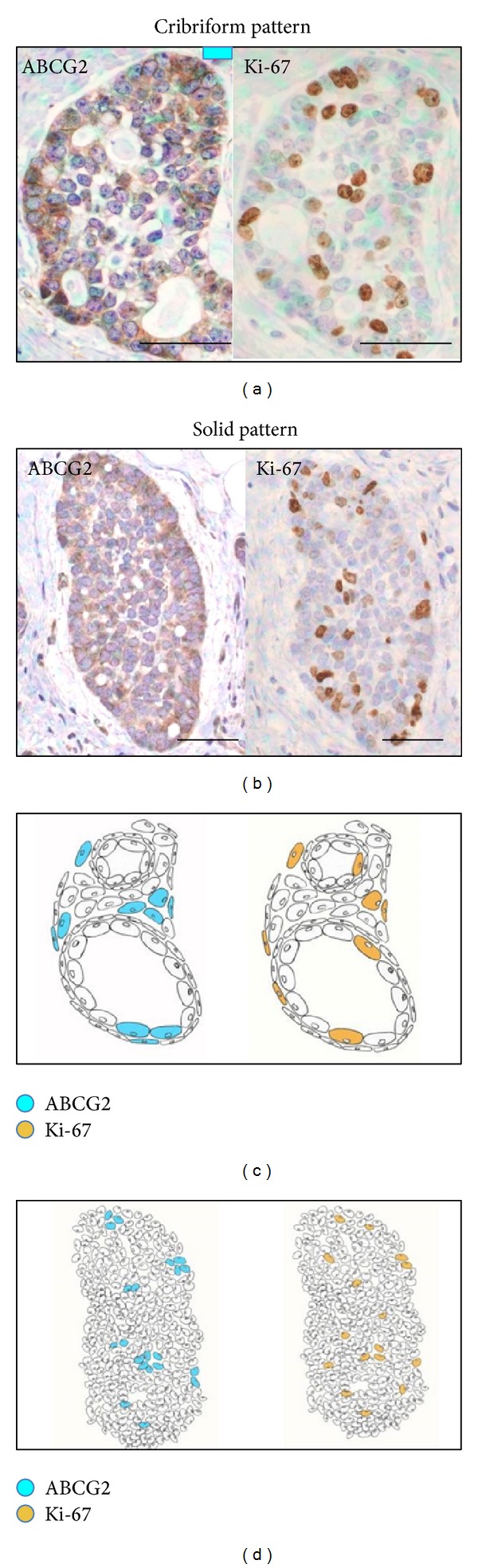
Positive expressions of ABCG2 and Ki-67 by the tumor cells were shown in the same area. The double positive stained cells were concentrated mainly in the peripheral zone of the cribriform pattern and also in the periphery and central zones of the solid pattern. Only a few strongly positive ABCG2 cells were negative for Ki-67 (scale bar: 50 *μ*m).

*Ki-67.* Positive expressions of Ki-67 were found in 15 ACC samples including 4 cases with weak staining intensity, 3 cases with moderate intensity, and 8 cases with strong intensity. The average positive percentage was about 60% (*P* = 0.001), which was significantly stronger compared to the normal looking tissues (*P* = 0.001). The Ki-67-positive stainings separated broadly in the tumor tissues of cribriform (Figure 2(d)) and solid pattern (Figure 4(d)). We also noticed that most cells overlaid with ABCG2 and Ki-67 were mainly found in the same location of the tumor tissue. A few cells expressing high level of ABCG2 with Ki-67-negative expression were surrounded by cells expressing both ABCG2 and Ki-67 (Figure 5).

### 3.3. Statistical Results

The distribution correlation of ABCG2 and Ki-67 in ACC was summarized in Figure 5. We also compared the expression correlations of different markers between ACC and normal looking tissues (Table 2). To further identify the expression correlations among ABCG2, CD133, podoplanin, and Ki-67 in normal looking tissues and ACC, we summarized the expression patterns of each marker in Tables 3 and 4. In normal looking tissues, there were no significant correlations between the two markers (*P* > 0.05) (Table 3). In ACC, only ABCG2 and Ki-67 showed a significant correlation (*r*_*s*_ = 0.620  and  *P* = 0.001) and no other significant associations were found between any two of the four markers (*P* > 0.05) (Table 4).

## 4. Discussion

To date, growing evidence has identified that CSC is the driving force of tumor initiation and progression in several tumor types [36, 37]. If CSCs can be identified and isolated from tumors using specific markers, new therapies may be established by effectively targeting the CSCs and therefore may improve the clinical outcomes of cancer treatment.

In this study, we characterized the expression patterns of ABCG2, CD133, and podoplanin in ACC of minor salivary glands by immunohistochemistry staining. We found a significantly higher expression of ABCG2 in ACC than in normal looking salivary gland tissues. We also exploited Ki-67 to detect the proliferative activities of tumor cells in ACC, and we noticed that ABCG2 was also expressed in the same location with Ki-67-positive cells. These overlaid cells were mainly located in the peripheral zone of the cancer nest in cribriform pattern and in the periphery and central regions of the cancer nest in solid subtype. More interestingly, we noticed that a few cells with strong staining of ABCG2 but negative staining with Ki-67 were usually surrounded by the cells expressing both ABCG2 and Ki-67. ABCG2 is widely expressed in many normal vital organs such as liver, lung, spleen, kidney, brain, nerve, skeletal muscle, cardiac muscle, testis, pancreas, small intestine, and trachea and also in a variety of tumors without tissue specificity [38, 39]. Meanwhile, ABCG2 shows dominant membrane staining in a wide range of stem cells [40] and is recognized as a universal marker for stem cells [41]. In this study, we found a diffusion expression pattern of ABCG2 in ACC and the expression level in ACC is significantly higher than that in normal looking tissues which suggested that ABCG2 might be involved in the development process of ACC and played a role in enhancing the proliferation and invasion capabilities of cancer cells. Compared to the expression pattern of Ki67, we also noticed that most ABCG2-positive cells also had positive staining with Ki-67 and mainly located in the periphery of cribriform cancer nests and in both periphery and central zones of solid cancer nests.

It is known that Ki-67, as a marker of cell proliferation, is absent in quiescent cells but has universal expression in proliferating cells [42]. According to the CSC theory, CSCs are rare and quiescent and have the potential of self-renewing and maintaining tumor growth and heterogeneity [43]. Some studies also reported that the Ki-67-positive cells are less likely to contain cancer stem-like cells due to their high degree of proliferation and differentiation activity [41]. Conversely, ABCG2 is found to be overexpressed in SP stem cells isolated form bone marrow [44] and a number of established cancer cell lines as well as tumor samples [45–47]. ABCG2 is also speculated to be critical in maintaining a quiescent state of the stem cells although the mechanism is still unclear [48]. From our results, a small number of cells with the ABCG2 strongest staining and Ki-67-negative staining (ABCG2+++/Ki-67−) could be repetitively found to be surrounded with cells expressing both ABCG2 and Ki-67. We speculate that cancer stem-like cells may exist in the cells with ABCG2+++/Ki-67− staining, which are surrounded by cells expressing both ABCG2 and Ki-67 in ACC and ABCG2 may play a role in the process of cellular proliferation and development in ACC. Our findings suggest that costaining with ABCG2 and Ki-67 may help predict the location of CSCs in ACC. Besides, the statistical analysis based on the total cases of ACC analyzed in this study showed a significant correlation between ABCG2 and Ki-67, which can further support our above findings. However, our sample size is not big enough for the statistical analysis according to the sample varies based on the clinical and histopathological classifications. More samples are needed in the future studies to identify the above findings based on sample clinical and histopathological varies and additionally determine the mechanism of their correlations.

We also explored the expression patterns of ABCG2, CD133, and podoplanin in normal looking and ACC tissues. We found that ABCG2 and CD133 had a very similar positivity in ACC (92% and 100%, resp.); however, the positive staining distributions of ABCG2 and CD133 in ACC were different. In the cribriform pattern of ACC, ABCG2 stainings were observed in cytoplasm and membrane of epithelial cells. In the tubular pattern, ABCG2 was expressed in glandular epithelial cells. In the solid pattern, the myoepithelial cells were diffusely positive for ABCG2. CD133 staining could only be found in the luminal cells of cribriform and tubular patterns. In the solid pattern, CD133-positive expression was mainly diffused among the dense myoepithelial cells. There was no significant correlation between CD133 and ABCG2 or Ki-67 based on their staining intensity and location. CD133 has been studied in recent years as a specific surface molecule in detecting CSCs and CD133-positive cancer cells which are also found possessing many stem cell characteristics [7, 25, 49]. Its attentiveness as a cancer stem cell marker has recently grown dramatically in breast cancer [17, 50], brain cancer [21], prostate cancer [22], pancreatic cancer [22], kidney cancer [51], and also in hepatocarcinoma [25] and melanoma [52]. In our study, we also noticed a similar expression pattern of CD133 in the normal looking tissues of human minor salivary glands as reported in the mammary glands, pancreas, and liver [53]. However, the CD133 expression pattern and intensity were similar in both normal looking tissues and in ACC from our study so that no definitive CD133-positive tumor cells could be distinguished in our ACC samples.

Podoplanin has also been investigated for its potential as a CSC marker in the solid tumors in recent studies. Podoplanin is found in various neoplasms, such as squamous-cell carcinoma [54], germ cell tumors [55], mesothelioma [56], and some subtypes of vascular tumors. Podoplanin is also considered to be involved in the process of tumor cell migration, invasion, and metastasis. From our results, podoplanin only exhibited weak positive staining in the cytoplasm and cell membrane of myoepithelial cells of normal looking tissues and cribriform and tubular patterns of ACC, but showed negative staining in solid pattern of ACC. Furthermore, we did not find any significant difference of podoplanin expressions between the normal looking tissues and ACC. All these results are not sufficient enough to support that CD133 or podoplanin can detect cancer stem cells in ACC. We speculate that a more accurate and specific way to quickly locate and identify the CSCs with clinical carcinoma samples is to target the candidate cells by costaining with multiple CSC markers. If CSCs can be successfully located, anticancer drugs can be precisely delivered into the cancer nest through tissue engineering technologies and the efficiency of the current clinical treatments can be significantly improved. Therefore, finding out the expression patterns and correlations of the CSC surface markers with immunohistological studies is an important and initial step towards this goal. In our study, we did not find out any significant associations among the investigated markers (ABCG2, CD133, and podoplanin) in ACC. More efforts will be put in exploring the expression patterns and correlations of other commonly used CSC markers such as CD44, CD24, CD29, CD90, and aldehyde dehydrogenase 1 (ALDH1) in ACC of minor salivary glands. Still, whether CD133 or podoplanin can be selected as a specific marker of CSCs in ACC and its biological and functional contribution to tumorigenesis needs to be answered in further investigations.

## 5. Conclusion

In conclusion, the expression of ABCG2 showed more significant upregulation in ACC than in normal looking tissues and it showed a similar expression pattern with Ki-67 in ACC. We speculate that the cancer stem-like cells may exist with ABCG2+++/Ki-67− staining, which are surrounded by cells with positive expression of ABCG2 and Ki-67 in ACC. Costaining with ABCG2 and Ki-67 may help predict the location of CSCs in ACC.

In future studies, we will study more clinical samples based on clinical and histopathological classification to find out the relationships between the expression level of ABCG2 and other CSC markers and the prognosis and clinical implications of ACC patients. We will explore the expression patterns and correlations of other surface markers that are commonly used in identifying CSCs such as CD44, CD24, CD29, CD90, and aldehyde dehydrogenase 1 (ALDH1) in ACC of minor salivary glands.

## Figures and Tables

**Table 1:** Clinical and histopathological data of 25 patients with ACC.

Number	Gender	Age (years)	Primary site	Histological pattern
1	F	78	Mouth floor	Cribriform
2	F	62	Mouth floor	Cribriform
3	F	42	Mouth floor	Cribriform
4	F	70	Mouth floor	Cribriform
5	F	78	Mouth floor	Cribriform
6	F	65	Mouth floor	Cribriform
7	F	58	Mouth floor	Cribriform with tubular
8	F	66	Mouth floor	Cribriform with tubular
9	M	67	Mouth floor	Cribriform with solid
10	F	46	Mouth floor	Cribriform with solid
11	M	57	Mouth floor	Cribriform with solid
12	M	75	Palate	Cribriform
13	M	77	Palate	Cribriform
14	M	62	Palate	Cribriform
15	F	71	Palate	Cribriform
16	F	66	Palate	Cribriform
17	F	34	Palate	Cribriform
18	F	71	Palate	Cribriform
19	F	32	Palate	Cribriform
20	M	54	Palate	Cribriform with tubular
21	M	53	Palate	Cribriform with tubular
22	F	51	Palate	Cribriform with tubular
23	M	52	Palate	Cribriform with solid
24	M	65	Buccal mucosa	Cribriform
25	F	66	Buccal mucosa	Cribriform

**Table 2:** Expressions of ABCG2, CD133, podoplanin, and Ki-67 in normal looking tissues and ACC.

Groups	*n*	ABCG2 intensity				Positivity (%)	CD133 intensity				Positivity (%)	Podoplanin intensity				Positivity (%)	Ki-67 intensity				Positivity (%)
		−	+	++	+++		−	+	++	+++		−	+	++	+++		−	+	++	+++	
Normal	10	4	6	0	0	60	0	0	5	5	100	9	1	0	0	10	10	0	0	0	0
ACC	25	2	1	7	15	92	0	1	12	12	100	19	4	1	1	24	10	4	3	8	60
*P*		0.000*				0.043*	0.835				1.000	0.332				0.644	0.001*				0.001*

Normal: normal looking tissues; Intensity: − negative, + weak staining, ++ moderate staining, and +++ strong staining.

*denotes significant difference when compared with the normal looking tissue (*P* < 0.05).

**Table 3:** The correlation information of ABCG2, CD133, and podoplanin in normal looking tissues.

	CD133		Podoplanin	
	*r*_*s*_	*P*	*r*_*s*_	*P*
ABCG2	0.298	0.403	0.124	0.732
CD133	—	—	−0.333	0.347

**Table 4:** The correlation information of ABCG2, CD133, podoplanin, and Ki-67 in ACC.

	CD133		Podoplanin		Ki-67	
	*r*_*s*_	*P*	*r*_*s*_	*P*	*r*_*s*_	*P*
ABCG2	−0.320	0.119	0.155	0.459	0.620	0.001*
CD133	—	—	0.237	0.254	−0.340	0.096
Podoplanin	—	—	—	—	0.069	0.743

*denotes significant difference (*P* < 0.05).
